# Oxidation-induced nanolite crystallization triggered the 2021 eruption of Fukutoku-Oka-no-Ba, Japan

**DOI:** 10.1038/s41598-023-34301-w

**Published:** 2023-05-09

**Authors:** Kenta Yoshida, Akira Miyake, Shota H. Okumura, Hidemi Ishibashi, Satoshi Okumura, Atsushi Okamoto, Yasuhiro Niwa, Masao Kimura, Tomoki Sato, Yoshihiko Tamura, Shigeaki Ono

**Affiliations:** 1 Research Institute for Marine Geodynamics, Japan Agency for Marine-Earth Science and Technology, Natsushima-cho 2-15, Yokosuka, 237-0061 Japan; 2 Department of Geology and Mineralogy, Kyoto University, Kitashirakawa-Oiwakecho, Sakyo-ku, Kyoto, 606-8502 Japan; 3 Department of Geoscience, Faculty of Science, Shizuoka University, Ohya 836, Suruga-ku, Shizuoka, 422-8529 Japan; 4 Division of Earth and Planetary Materials Science, Department of Earth Science, Graduate School of Science, Tohoku University, 6-3 Aramaki-Aza-Aoba, Aoba-ku, Sendai, Miyagi 980-8578 Japan; 5 Graduate School of Environmental Studies, Tohoku University, Sendai, Miyagi 980-8579 Japan; 6 Institute of Materials Structure Science, High Energy Accelerator Research Organization, 1-1 Oho, Tsukuba, Ibaraki 305-0801 Japan

**Keywords:** Geochemistry, Petrology, Volcanology

## Abstract

Nanometer-sized crystals (nanolites) play an important role in controlling eruptions by affecting the viscosity of magmas and inducing bubble nucleation. We present detailed microscopic and nanoscopic petrographic analyses of nanolite-bearing and nanolite-free pumice from the 2021 eruption of Fukutoku-Oka-no-Ba, Japan. The nanolite mineral assemblage includes biotite, which is absent from the phenocryst mineral assemblage, and magnetite and clinopyroxene, which are observed as phenocrysts. The boundary between the nanolite-bearing brown glass and nanolite-free colorless glass is either sharp or gradational, and the sharp boundaries also appear sharp under the transmitted electron microscope. X-ray absorption fine structure (XAFS) analysis of the volcanic glass revealed that the nanolite-free colorless glass records an oxygen fugacity of QFM + 0.98 (log units), whereas the nanolite-bearing brown glass records a higher apparent oxygen fugacity (~ QFM + 2). Thermodynamic modelling using MELTS indicates that higher oxygen fugacities increase the liquidus temperature and thus induced the crystallization of magnetite nanolites. The hydrous nanolite mineral assemblage and glass oxygen fugacity estimates suggest that an oxidizing fluid supplied by a hot mafic magma induced nanolite crystallization in the magma reservoir, before the magma fragmentation. The oxidation-induced nanolite crystallization then enhanced heterogeneous bubble nucleation, resulting in convection in the magma reservoir and triggering the eruption.

## Introduction

Nanoscale crystals, known as nanolites, play an important role during eruptions. Nanolites were originally distinguished from microlites by a pronounced break in the crystal size distribution (CSD) at < 600 nm^1^, and Mujin et al.^2^ later redefined nanolites as crystals of 30–1000 nm in length and ultrananolites as crystals of < 30 nm in length. Conventional petrographic studies of nanolites have required high-resolution observation systems, for example, the transmission electron microscope (TEM) or high-resolution-scanning electron microscope (HR-SEM). Raman microscopy has made the detection of Fe–Ti oxide (magnetite) nanolites increasingly easy^3,4^. The crystallization of nanolites is generally thought to reflect shallow processes, including magma ascent in the conduit^4–8^ and the cooling process after fragmentation of magma^9^. It has also been shown that the crystallization of magnetite nanolites may enhance the explosivity of an eruption by increasing the viscosity of the magma or increasing bubble nucleation^4,10–13^. However, recent in situ experiments indicate that although nanolite crystallization increases the viscosity, the increase effect in the natural melt is not as high as expected from analogue materials^14^, and the relationship between nanolites and volcanic processes remains unclear. Furthermore, how nanolite crystallization (or bubble nucleation) begins in erupting magma remains unclear.

Fukutoku-Oka-no-Ba (FOB) is a submarine volcano in the Izu–Ogasawara arc in the northwest Pacific, ~ 1300 km south of mainland Japan (24°17.1′N, 141°28.9′E). The summit of the volcano has a flat oval shape with the length of 1.5 × 1 km at the depth of ~ 30 m below sea level before the 2021 eruption^15^. On 13–15 August 2021 (Japan Standard Time), there was an explosive eruption at the volcano^16–18^. Based on the satellite observation, Maeno et al.^16^ indicated the eruption column was water-rich with small amount of volcaniclastic materials, and thus, the explosivity of the eruption was increased by an interaction between seawater and a high magma discharge rate. The eruption produced a large pumice raft, mainly consisting of gray-colored pumice, which was carried westward by ocean currents for > 1000 km^18,19^. The pumice raft arrived first on the Pacific coasts of the Japanese islands and subsequently traveled west for a total of > 5000 km, arriving in the Gulf of Thailand^20^. Large amounts of floating pumice can damage coastal ecosystems and impact the economy^16,18,21^. Geochemical and petrological analyses of the drift pumice showed that despite their variable colors (gray, amber, brown, and black), they have almost homogeneous trachytic compositions with SiO_2_ and Na_2_O + K_2_O contents of 60–65 and 8–10 mass%, respectively^18^. Although the deposited pumice clasts have undergone several abrasion and elimination process during drifting for > 1000 km and for 2 months, the overall trend of the pumice type, i.e., the majority is gray type, remained same compared to that observed within 10 days after the eruption on the sea^16,19^. The pumice of different colors occurs either independent clasts or together in a single clast with gradual or sharp boundaries. A notable characteristic of the FOB pumice is the common occurrence of small volumes of black pumice while the majority was gray pumice. The black pumice has a similar composition to the gray pumice that makes up most of the deposit, although they have different microtextures. Raman microscopy showed that the brown glass in the black pumice contains magnetite nanolites that increased the melt viscosity and thus played a role in the explosive 2021 FOB eruption^18^.

We performed a comprehensive study of the nanolite-bearing glass in the FOB pumice, including TEM analysis, Fe K-edge XANES (X-ray absorption near edge structure) microanalysis, and thermodynamic modelling. The investigated sample (AYA-2) is a single pumice clast composed of gray and black parts with a sharp boundary and was collected from the northeastern coast of Amami Ōshima (28°28.4′N, 129°42.9′E) on October 18th, 2021 (Supplementary Fig. 1). Basic petrographic descriptions of this sample have been presented^18^. We also discuss how nanolite crystallization occurred in the FOB magma reservoir and affected the eruption.

## Results

### Petrography

The pumice raft from the 2021 FOB eruption consists mostly of gray pumice with a small amount of black and different colored (including amber and brown) pumice. The black and brown pumice consist of brown glass with a magnetite nanolite Raman signature, with a peak^18^ at ~ 670 cm^−1^. In contrast, the gray and amber pumice are composed of colorless and nanolite-free glass. Visible microlites have not been identified in the gray pumice, whereas the black pumice contains rare clinopyroxene and olivine microlites^18^. The black pumice occurs either as individual clasts or mingled with the gray pumice^18,20^.

**Figure 1:**
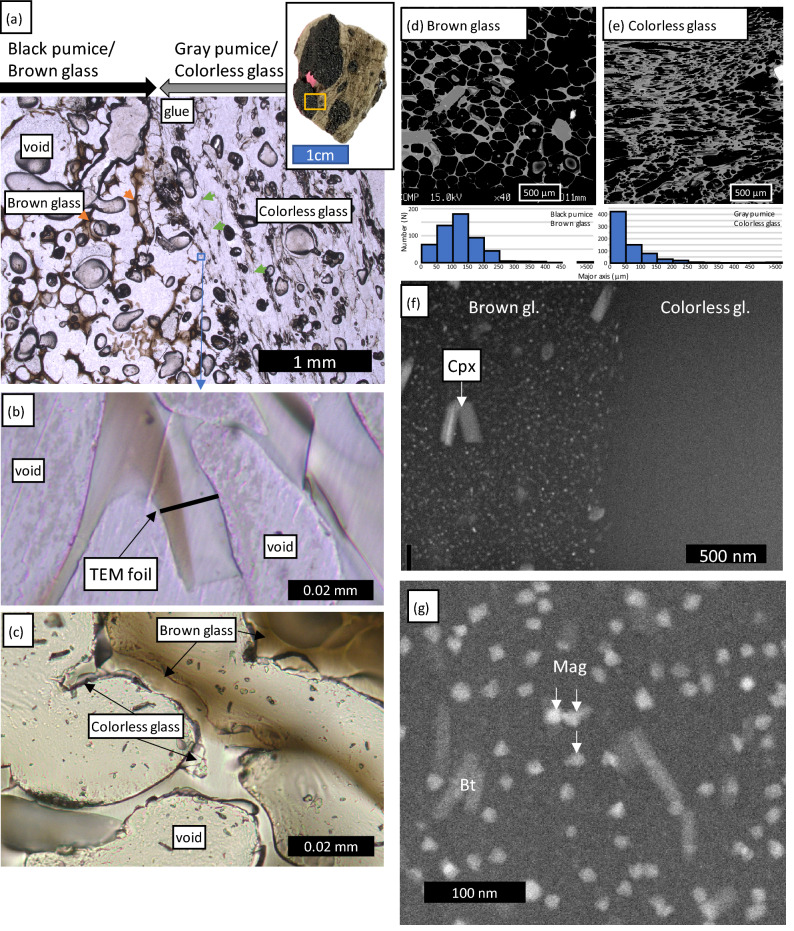
(**a**) Photomicrograph of the boundary between the black pumice (brown glass) and gray pumice (colorless glass). The blue box indicates the position of the enlarged photomicrograph shown in (**b**). The inset photograph shows the whole clast. (**b**) Enlarged view of the boundary between the brown and colorless glasses. The black line indicates the position of the TEM analysis. (**c**) Gradational boundary between the brown and colorless glass in a different pumice clast. (**d**,**e**) Representative backscattered electron images of the (**d**) brown and (**e**) colorless glass domains. The brown glass has larger, spherical bubbles, whereas the colorless glass has small, elongated bubbles. Histograms of the major axis lengths of the bubbles are also shown. (**f**) TEM bright field image of the area indicated in (**b**). The boundary between the brown and colorless glasses is clear at this scale. Relatively large clinopyroxene nanolites are seen. (**g**) Enlarged view of the brown glass, showing magnetite nanolites ~ 20 nm in length and biotite nanolites ~ 100 nm in length. Abbreviations are clinopyroxene (Cpx), biotite (Bt), and magnetite (Mag).

The gray and black pumice often exhibit different textures (Fig. 1a,d,e). Smaller, more elongated vesicles were observed in the groundmass of the gray pumice, whereas those in the black pumice were larger and more spherical (Fig. 1d,e). The major axes of the bubbles were estimated using ellipsoid fitting. Although large bubbles (> 500 μm) were identified in both types of glass, most bubbles in the colorless glass were < 50 μm. The mean lengths of the bubbles in the colorless and brown glass were 73 and 128 μm, respectively. The boundaries between the gray and black pumice varied: some clasts contained sharp boundaries between the brown (black pumice) and colorless (gray pumice) glass under the optical microscope (Fig. 1b), whereas others exhibited a gradual change from the brown to colorless glass (Fig. 1c). Phenocryst mineral assemblages are similar in both pumice types (clinopyroxene, plagioclase, and minor magnetite and olivine), and most minerals have similar compositions, except for those that likely originated in a mafic magma^18,20^. For example, two types of olivine are observed in the FOB pumice: one relatively Fe-rich (Mg# = molar Mg/[Mg + Fe] ~ 65) without compositional zonation and one with a high-Mg (Mg# ~ 90) plateau and decreasing Mg contents toward the rims^18^. The latter type of olivine is observed in or closely associated with the black pumice^18,20^.

We performed TEM analyses on the sharp boundary between the two types of glass to identify the differences between the brown nanolite-bearing glass and the colorless nanolite-free glass (Fig. 1b).

### TEM analysis

TEM analysis revealed three types of nanolite in the brown glass. In contrast, the colorless glass was crystal-free even at the scale of TEM analysis (Fig. 1f). The largest grains were clinopyroxene, with long axes of < 300 nm. In contrast, the abundant < 20 nm blocky grains were magnetite (Fig. 1g). Occasional tabular grains < 100 nm in length were observed, which yielded K, Al, and Mg EDS signatures, suggesting that they were biotite.

The magnetite nanolites were randomly orientated; however, the elongated clinopyroxene and biotite grains were weakly aligned (sub)parallel to the boundary between the brown and colorless glass. The nano-scale solid phase was ~ 12 vol% of the sample based on the TEM image (Fig. 1g).

### XANES analyses

**Figure 2:**
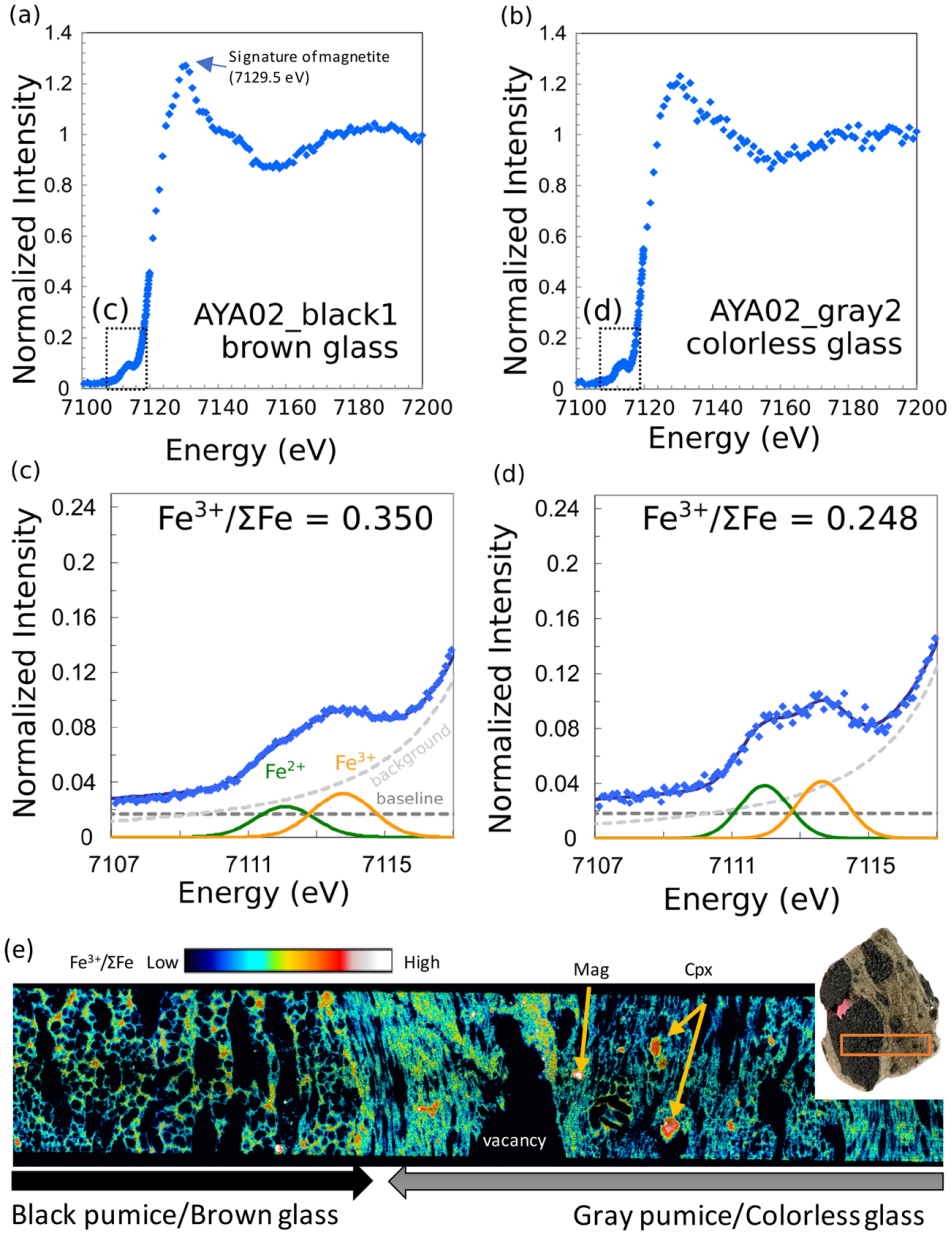
Representative XANES spectra from the (**a**) brown and (**b**) colorless glasses. (**c**,**d**) Enlarged view of the pre-edge region indicated by the dashed box in (**a**) and (**b**). Gaussian fits for the Fe^2+^ and Fe^3+^ peaks are also shown. Fe^3+^/ΣFe ratios were calculated using the calibration for rhyolite glass of Okumura et al.^39^. (**e**) 2D XANES image of the boundary between the black and gray pumice domains. The analyzed thick section was made from the clast used for the spot analysis. Areas with higher Fe^3+^/ΣFe ratios are clinopyroxene and magnetite phenocrysts, indicated by arrows.

Representative XANES spectra obtained by spot analyses of the colorless and brown glasses are shown in Fig. 2a–d with the calculated Fe^3+^/ΣFe ratios. The Fe^3+^/ΣFe ratios of the colorless and brown glasses were 0.24–0.28 (n = 4) and 0.31–0.36 (n = 8), respectively. In addition, the XANES spectra of the brown glass had a relatively sharp peak at ~ 7129.5 eV that can be attributed to the magnetite^22^, indicating that we analyzed the brown glass as the mixture of nanolite and amorphous silicate glass. The presence of magnetite nanolites may invalidate the Fe XANES centroid energy used in the calibration of the Fe^3+^/ΣFe ratio; therefore, these values should be interpreted with caution. Although the true Fe^3+^/ΣFe ratio of the amorphous part in the brown glass is uncertain, it should be noted that the entire mixture of the brown glass, i.e., nanolite + amorphous part, is rich in Fe^3+^ and is more oxidized than the colorless nanolite-free glass.

2D XANES analysis also showed that the brown glass in the black pumice had higher Fe^3+^/ΣFe ratios than the colorless glass in the gray pumice (Fig. 2e).

### MELTS modeling

Although the timescale of nanolite-formation would be short and the metastable or disequilibrium process can be expected, the thermodynamic phase modelling can be used as a useful indicator to consider its formation process. The stable mineral assemblage for the FOB pumice composition was calculated using rhyolite-MELTS v.1.2.x model^23^. The FOB pumice has a narrow range of whole-rock compositions, despite its appearance^18^. The whole rock composition of FOB-JMA-18^18^ was used in the modelling.

The oxygen fugacity (*f*O_2_) of the colorless and brown glasses were calculated using the formula of^24^, the composition of FOB-JMA-18, and the reported pressure and temperature of the magma reservoir (930 °C and 250 MPa^18^). Under these conditions and with the measured Fe^3+^/ΣFe ratio, the log(*f*O_2_) values of the colorless glass relative to the QFM (quartz–fayalite–magnetite) buffer is QFM + 0.98. Although the XANES spectra of the brown glass includes a signal from magnetite nanolites, we use the apparent Fe^3+^/ΣFe ratio to calculate a *f*O_2_ of QFM + 2.04 for the brown glass.

**Figure 3:**
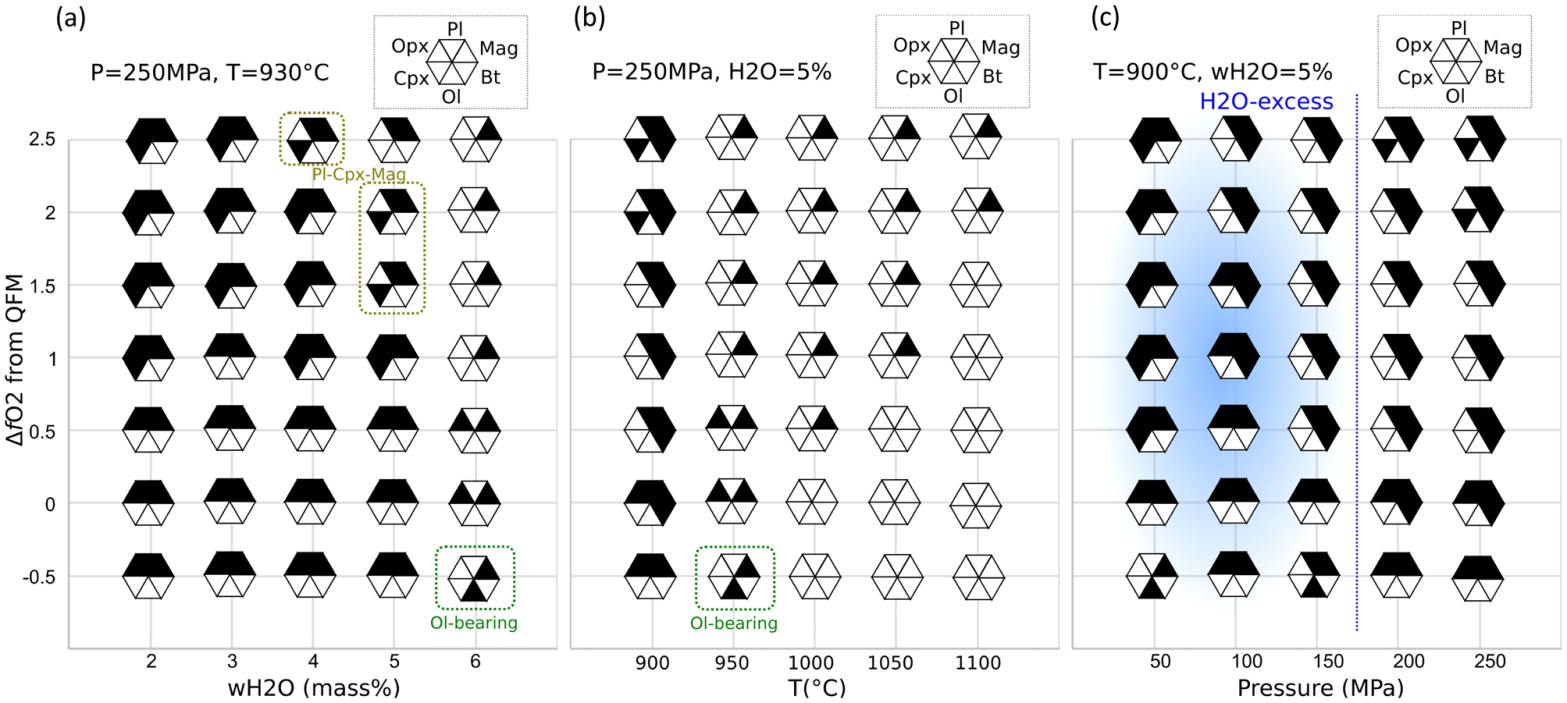
Stable phases according to MELTS_Excel at (**a**) fixed pressure and temperature and variable oxygen fugacity and water contents, (**b**) fixed pressure and water content (5 mass%) and variable oxygen fugacity and temperature, and (**c**) fixed temperature and water content (5 mass%) and variable oxygen fugacity and pressure. Abbreviations are plagioclase (Pl), orthopyroxene (Opx), clinopyroxene (Cpx), olivine (Ol), biotite (Bt), and magnetite (Mag).

To model the appearance of nanolites and phenocrysts in the magma reservoir, we used a fixed temperature of 930 °C and a pressure of 250 MPa and changed the *f*O_2_ and water content, as summarized in Fig. 3a. Magnetite is stable under all modelled conditions. Olivine, with a Mg# of ~ 60, was found to be stable only under reduced (QFM − 0.5) and wet (H_2_O = 6 mass%) conditions, whereas the other phenocryst minerals (clinopyroxene, plagioclase, and magnetite) were stable under more oxidized (QFM + 1.5 and + 2) conditions with relatively high water contents (5 mass%).

We also modelled the phase relationships with changing temperature and *f*O_2_ at a constant pressure of 250 MPa and a fixed water content of 5 mass% (Fig. 3b). The liquidus temperature reaches > 1100 °C at QFM + 2, whereas more reduced conditions yield lower temperatures (< 1000 °C). Biotite crystallizes at relatively high *f*O_2_ (> QFM + 0) and low temperatures (< 925 °C).

To further evaluate the stability of biotite, we modelled variable pressures and *f*O_2_ at a constant temperature of 900 °C and a water content of 5 mass% (Fig. 3c). At low pressures (< 150 MPa), H_2_O becomes saturated. Biotite becomes stable at higher pressures (> 100 MPa) and *f*O_2_ (> QFM + 1). The required oxygen fugacity for the biotite stability becomes lower at higher pressures.

## Discussion

The XANES analyses showed that the difference between the gray and black pumice, the occurrence of nanolites, can be attributed to a difference in the Fe^3+^/ΣFe ratios and the corresponding *f*O_2_. The brown glass with magnetite nanolites occurs in the domain with high apparent *f*O_2_ (~ QFM + 2.04), whereas the colorless glass domains yield an *f*O_2_ of ~ QFM + 0.98. Although there is uncertainty in the calibration of the Fe^3+^/ΣFe for nanolite-bearing glass^22^, the brown glass has a higher Fe^3+^/ΣFe ratio and thus experienced a higher *f*O_2_. This study focused on the most typical nanolite-free and nanolite-bearing samples; i.e., the boundary between gray and black pumice. The sharp boundary between the nanolite-bearing brown glass and nanolite-free colorless glass (Fig. 1b) was formed by a rapid process, for example, the mingling of two types of magma during an explosive eruption, suggesting that the two magmas were different colors before the eruption.

The textures of the bubble in the two domains are different, with small, elongated bubbles in the colorless glass and large, spherical bubbles in the brown glass (Fig. 2d,e). These contrasting textures and their relationship with the presence of nanolites suggests that bubble nucleation occurred earlier in the brown glass than in the colorless glass, allowing the bubbles to mature. Bubble nucleation began subsequently in the nanolite-free magma, possibly after the two magmas mingled. This scenario can be best explained by the triggering of bubble nucleation by the crystallization of nanolites prior to eruption^10,25^, rather than by bubble-induced nanolite crystallization^12^. It should be noted that Kato^26^ studied light and dark gray pumice from the 1986 FOB eruption and showed that the two types of pumice had the same Fe^2+^/Fe^3+^ ratio using titration. Based on the descriptions of the dark gray pumice by^26^, including bubble microtextures, it may be the same as the amber pumice described by Yoshida et al.^18^ and different from the black pumice studied here. The amber pumice consists of colorless glass with relatively large vesicles and is free of magnetite nanolites^18^.

The experimental study in sulfur-free rhyolite system indicated that the degassing of H_2_O-dominated volatile component produces an increase in Fe^3+^/ΣFe^27^, although both black and gray types in the FOB pumice have undergone degassing during the decompression and ejection process. Therefore, the degassing-related oxidation is not likely to be the origin of the black pumice and the nanolite-forming process have occurred before starting of the eruption. The presence of high-Mg olivine associated with the black pumice suggested that the high-Mg olivine originated from hot mafic magma from depth that triggered the explosive eruption and that the black pumice (magma) had become black (nanolite-bearing) because of the effect of the intruded hot mafic magma^18^. The whole-rock composition of the black pumice is similar to that of the gray pumice, suggesting that instead a hot volatile component with small amount of solid was injected into the trachytic magma reservoir of FOB. The presence of biotite nanolites (Fig. 1e), as well as amphibole inclusions in high-Mg olivine^18^, suggests that this volatile component was water-rich. The MELTS modelling (Fig. 3c) suggests that the hydration and biotite formation did not occur at shallow depths, for example in the conduit, but in the deeper part (> 100 MPa) of the plumbing system. Adding water generally decreases the liquidus temperature; however, more oxidized conditions increase the liquidus temperature. The different Fe^3+^/ΣFe ratios of the gray and black pumice strongly suggest that the agent that darkened the black pumice was an oxidant. High water contents and *f*O_2_ are two fundamental characteristics of magmas formed in subduction zones and are acquired as hydrous primary melts react with the surrounding mantle^28^.

Given the contrasting textures of the brown and colorless glasses and the sharp boundary between them, the difference in the two magmas must have been generated before the eruption, possibly in the magma reservoir. The gradational boundaries between the brown and colorless glass (Fig. 1c) were formed by a slow process, for example the diffusive oxidation of the magma, or generated by the deformation of brown and colorless boundary during the eruption. The common occurrence of banded and mingled black and gray pumice^18,20^ also suggests nanolite crystallization in the magma reservoir. The experiments using mafic magmas showed that nanolite crystallization starts at higher temperatures but at a slower rate under oxidized conditions^29^, demonstrating that oxidation can promote the crystallization of nanolites.

**Figure 4:**
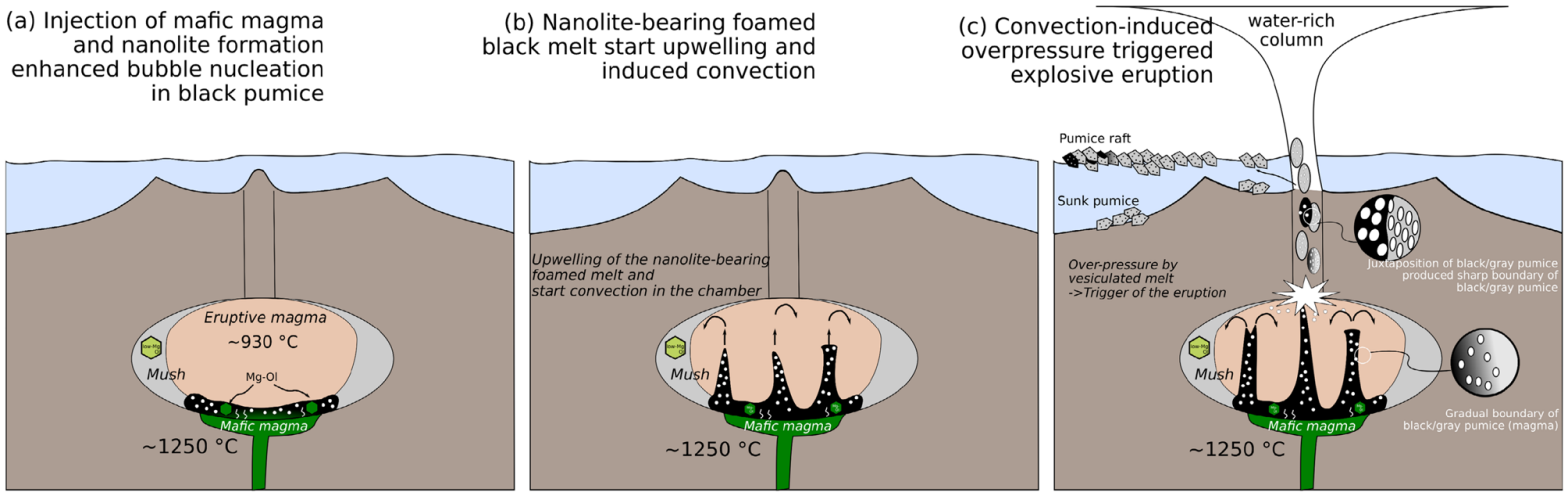
Pumice production during the 2021 FOB eruption. (**a**) The oxidizing agent and high-Mg olivine were supplied by hot mafic magma, which enhanced the crystallization of magnetite nanolites. Heterogeneous bubble nucleation started first in the nanolite-rich domain. (**b**) The bubbly magma became buoyant and initiated convection in the magma reservoir, which enhanced the rejuvenation of the crystal mush. (**c**) An eruption started when the critical pressure was produced by bubble nucleation in the rejuvenated magma.

The heating experiments under atmospheric conditions using rhyolitic pumice from the Havre volcano showed that the pumice heated for > 5 min at > 700 °C became pinkish owing to the oxidation of magnetite nanolites and their transformation into hematite^30^. Those authors suggested that the common occurrence of pink pumice in the 2012 Havre pumice raft evidenced that the water column of the explosive eruption was so powerful that the pumice had undergone high-temperature atmospheric iron oxidation. In contrast, oxidized pink pumice was not observed in the 2021 FOB pumice raft. The oxidation of iron sulfide to magnetite in the FOB pumice has been reported^18^, though further oxidation has not been observed. Satellite observations of a vigorous white plume during the 2021 FOB eruption suggests that it was a water-rich eruption, and the pumice raft was generated at a submarine vent^16^. The limited oxidation of the pumice is consistent with satellite observations. Also, the nanolite characteristics (Fig. 1d–g) are clearly different from those of the recycled ash that once have been ejected and fallen into the hot vent^31^. This suggests that the mingling of the black pumice with the gray pumice, which formed the sharp boundary between the brown and colorless glass, took place in the conduit (Fig. 4).

The formation of nanolites and related eruption processes can be summarized as follows (Fig. 4). Hot mafic magma (~ 1250 °C^18^) derived from the deeper part of the subduction zone is a clue to trigger of the eruption. Once the hydrous and highly oxidized primary magma was injected into the bottom of the FOB magma reservoir, it supplied an oxidizing fluid to the trachytic FOB magma. Oxidation-induced nanolite crystallization can enhance heterogeneous bubble nucleation^32–34^, and thus a portion of the oxidized melt in the magma chamber became buoyant. The bubbly nanolite-bearing melt started to ascend and initiated convection in the FOB magma reservoir, possibly inducing additional bubble nucleation in the other parts of the reservoir. Convection enhanced the rejuvenation of the magma reservoir, producing overpressure and triggering an eruption.

Paredes-Mariño et al.^35^ showed that macroscopic fragments of hotter magma injected into a magma reservoir provide sites for heterogeneous bubble nucleation and initiate convection. Although fragments of the injected mafic magma, such as black enclaves and high-Mg olivine, can be identified in the FOB pumice^18,20^, the higher Fe^3+^/ΣFe ratio of the brown glass strongly indicates that oxidation-induced nanolite crystallization played an important role in heterogeneous bubble nucleation. The sharp boundaries between the black and gray pumice formed by the coalescence of two kinds of pumice in the conduit during the eruption (Fig. 4). Contrasting microstructure of the black and gray pumice also indicates the coalescence of two pumice (magma) during or after bubbling. According to this scenario, bubble nucleation occurred earlier in the black pumice, while it was in the magma reservoir, as the magnetite nanolites provided nucleation sites^25^. In contrast, bubble nucleation occurred in the gray pumice during convection and its ascent up the conduit, consistent with the larger vesicles in the black pumice and smaller vesicles in the gray pumice (Figs. 1a, 2e).

A diffusion profile in a high-Mg olivine grain indicated that the mafic magma was injected into the reservoir before 14 h to 50 days of eruption, assuming it maintained its original temperature (~ 1250 °C)^18^. The viscosity of trachyte melt^36^ with ~ 12 vol.% nanoscopic crystals may be 10^4^–10^5^ Pa·s at ~ 900 °C, suggesting that the magma started convection ~ 10 days after the hotter magma was injected, if a simple two-layer model was assumed^37^. The oxidation-induced nanolite crystallization and bubble nucleation proposed here leads to increased convection compared with a simple heat convection model, i.e. the timescale of the convection can be shorter. Accordingly, the expected timescale for the initiation of magma convection, being several hours to days, might have preserved the nanolite precipitated in the black pumice.

## Conclusions

The 2021 FOB eruption produced a large volume of pumice, most of which was nanolite-free gray pumice, with minor nanolite-bearing black pumice. The occurrence of nanolite-free and nanolite-bearing pumice suggests the nanolites formed in the magma reservoir. XANES analyses and TEM observations show that the nanolite-bearing black pumice was oxidized and hydrated, suggesting that a flux of oxidized fluid from the underlying hot mafic magma induced the nanolite crystallization and heterogeneous bubble nucleation, which triggered the explosive eruption. Our study shows that oxidation by a fluid plays a vital role in nanolite crystallization.

## Methods

SEM analyses were performed using a field emission gun electron probe microanalyzer (JEOL JXA-8500F) at the Japan Agency for Marine–Earth Science and Technology, Yokosuka, Japan.

Nanolite analyses were conducted using a TEM (JEOL JEM-2100F) equipped with an energy-dispersive X-ray spectrometer (JEOL JED-2300T) at the Department of Geology and Mineralogy, Kyoto University, Japan. Before the TEM analyses, the thin foil the area of interest was cut out using a focused ion beam system (Thermo Scientific Helios Nanolab G3 CX) at the Department of Geology and Mineralogy, Kyoto University, Japan.

The Fe^3+^/ΣFe ratio of the glass was determined using Fe K-edge XANES spectra measured in fluorescence mode at room temperature using the BL-4A beamline at the Photon Factory, Tsukuba, Japan. The current of the X-ray storage ring was 450 mA. The X-rays were focused on a 6 × 4 μm area. The spectral features were deconvolved following the procedure of^38^, and the Fe^3+^/ΣFe ratios were calculated using the formula for rhyolitic glass^39^.

The two-dimensional distribution of the Fe^3+^/ΣFe ratio was qualitatively determined using Fe K-edge XAFS (X-ray Absorption Fine Structure) spectra measured using the NW2A beamline at the Photon Factory, following the procedure of^40^. The XAFS spectra at energies of 7076–7321 eV were measured in transmission mode using a 2048 × 1024 pixel detector and a spatial resolution of ~ 4.5 × 4.5 μm over an area of ~ 15 × 3 mm. The XAFS spectra were calibrated for the similarities to the reference endmembers of olivine (Fe^2+^) and andradite (Fe^3+^), and the calibrated values can be considered a qualitative measure of the Fe^3+^/ΣFe ratio.

Thermodynamic crystallization modelling was performed using MELTS_Excel^23^. Given that the reported mineral assemblage of the FOB pumice does not contain quartz, we used rhyolite-MELTS version 1.2.x.

## Supplementary Information

Supplementary Figure 1.

## Data Availability

All data generated or analyzed during this study are included in this published article and its supplementary information files.
