# Supplementary material for: Oxidation-induced nanolite crystallization triggered the 2021 eruption of Fukutoku-Oka-no-Ba, Japan
Source: Sci Rep. 2023 May 9;13:7117. doi: 10.1038/s41598-023-34301-w (PMC10170078; doi:10.1038/s41598-023-34301-w)
Supplement: Supplementary file 1 — Supplementary Figure 1. [file 41598_2023_34301_MOESM1_ESM.docx]

Supplementary Figure

Supplementary Figure 1. (a) Summary of the arrival day of the drift pumice from the 2021 FOB eruption modified after [1,2]. (b) Enlarged map of the position of sample collection, the northeastern coast of Amami Ōshima (28°28.4′N, 129°42.9′E). (c) The field view of the stranded pumice at the place shown in Figure Sb.

References:

1. Yoshida, K., Tamura, Y., Sato, T., Hanyu, T., Usui, Y., Chang, Q., Ono, S. Variety of the drift pumice clasts from the 2021 Fukutoku-Oka-no-Ba eruption, Japan. Island Arc, 31, e12441 (2022).
2. Yoshida, K., Tamura, Y., Sato, T., Sangmanee, C., Puttapreecha, R., & Ono, S. Petrographic characteristics in the pumice clast deposited along the Gulf of Thailand, drifted from Fukutoku-Oka-no-Ba. Geochemical Journal, 56(5), 134-137 (2022).
